# Going Full Circle with Organocatalysis and Biocatalysis: The Latent Potential of Cofactor Mimics in Asymmetric Synthesis

**DOI:** 10.1021/acs.joc.2c02747

**Published:** 2023-04-26

**Authors:** Jacob Murray, David R. W. Hodgson, AnnMarie C. O’Donoghue

**Affiliations:** Department of Chemistry, Durham University, South Road, Durham DH1 3LE, United Kingdom

## Abstract

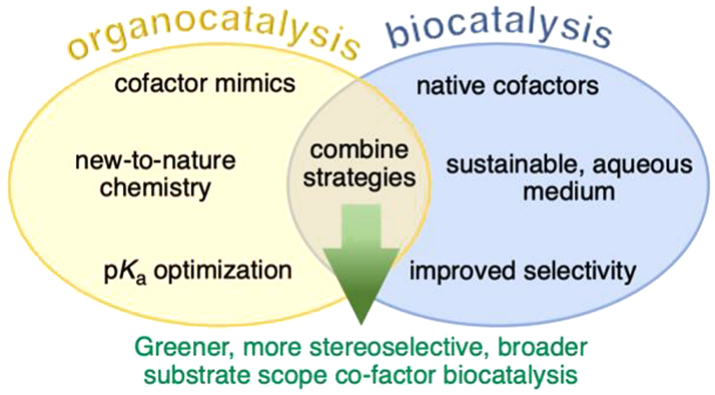

Many enzymes work in tandem with small molecule cofactors, which have inspired organocatalyst designs. Chemical modification of cofactor scaffolds has increased organocatalytic reactivity and reaction scope. This synopsis presents a selection of recent advances in the use of cofactors (native and mimics) in organocatalysis and biocatalysis. We aim to highlight the benefits of combining fundamental knowledge gained in both bio- and organo-catalysis for asymmetric biocatalysis.

## Introduction

**Scheme 1:**
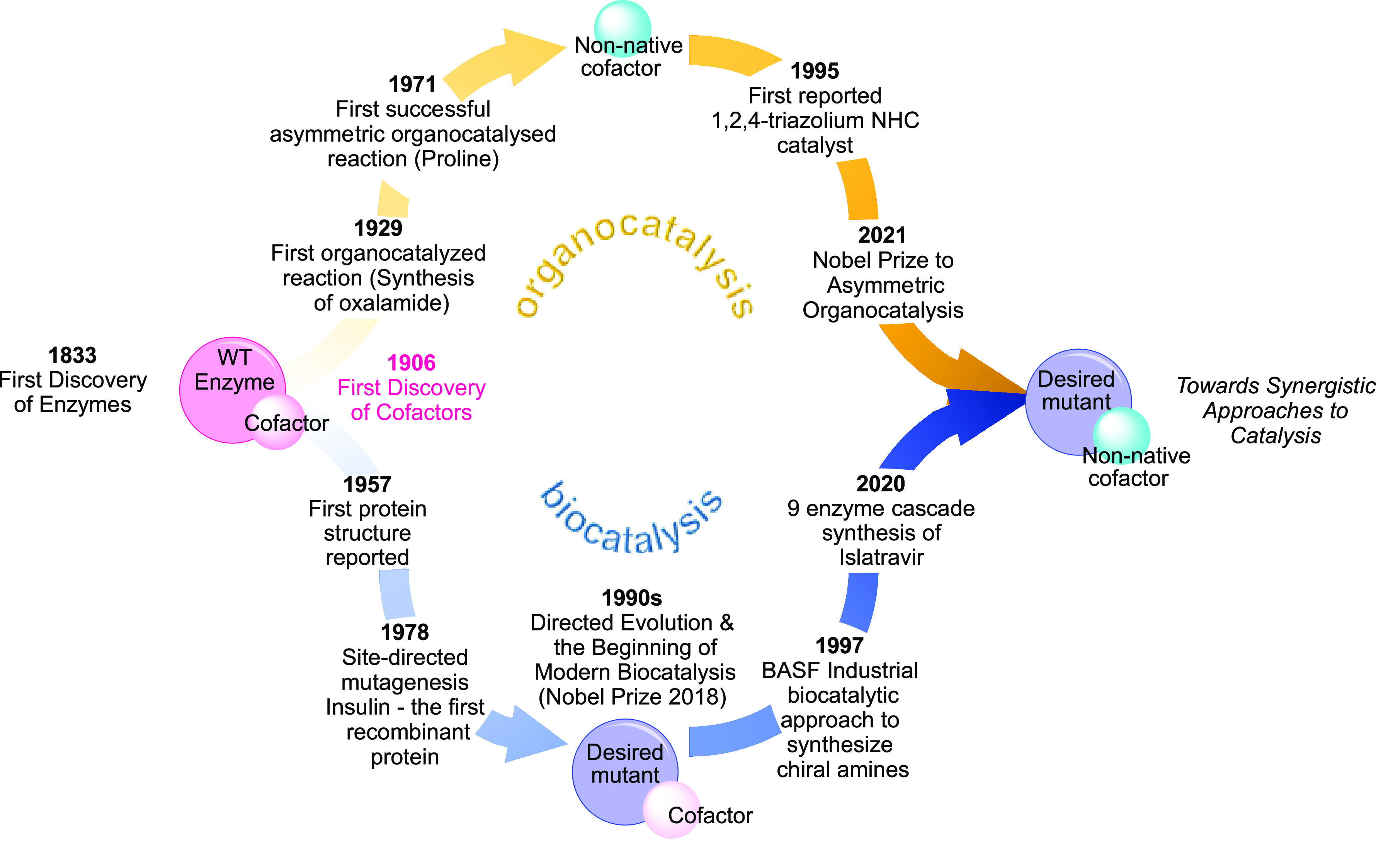
Timelines of Key Milestones in Organocatalysis and Biocatalysis

Often described as the perfect catalysts, enzymes have provided inspiration to much of organic chemistry, notably the field of organocatalysis. Although frequently considered as two distinct fields, biocatalysis and organocatalysis together offer many synergistic benefits and opportunities for catalysis (Scheme 1). Organocatalysis involves the use of small organic molecules to catalyze reactions.^1,2^ Asymmetric or enantioselective organocatalysis using chiral organocatalyst scaffolds has been the major focus of researchers with many examples of successful asymmetric syntheses employing this approach.^3,4^ Organocatalysis can require the potentially challenging syntheses of chiral catalysts but benefits from enhanced substrate scopes and greater accessibility to chemists. Biocatalysis approaches involving enzymes offer many advantages to chemical processes, operating under mild reaction conditions, in aqueous solvents to deliver complex molecular architectures with excellent stereoselectivities.^5,6^ With the imperative drive to “greener” chemical processes, the use of enzymes in catalysis is key to developing a sustainable future.^7^ In terms of limitations, wild-type enzymes do not usually possess the desired substrate scope or tolerance to reaction conditions to tackle the broad requirements of process chemistry. Furthermore, the natural repertoire of enzyme-catalyzed transformations does not include many reactions important to modern day synthetic chemistry. Major developments in protein engineering, such as directed evolution, have enabled access to enzymes with activities and specificities tailored to the desired chemical process.^8−13^

**Scheme 2:**
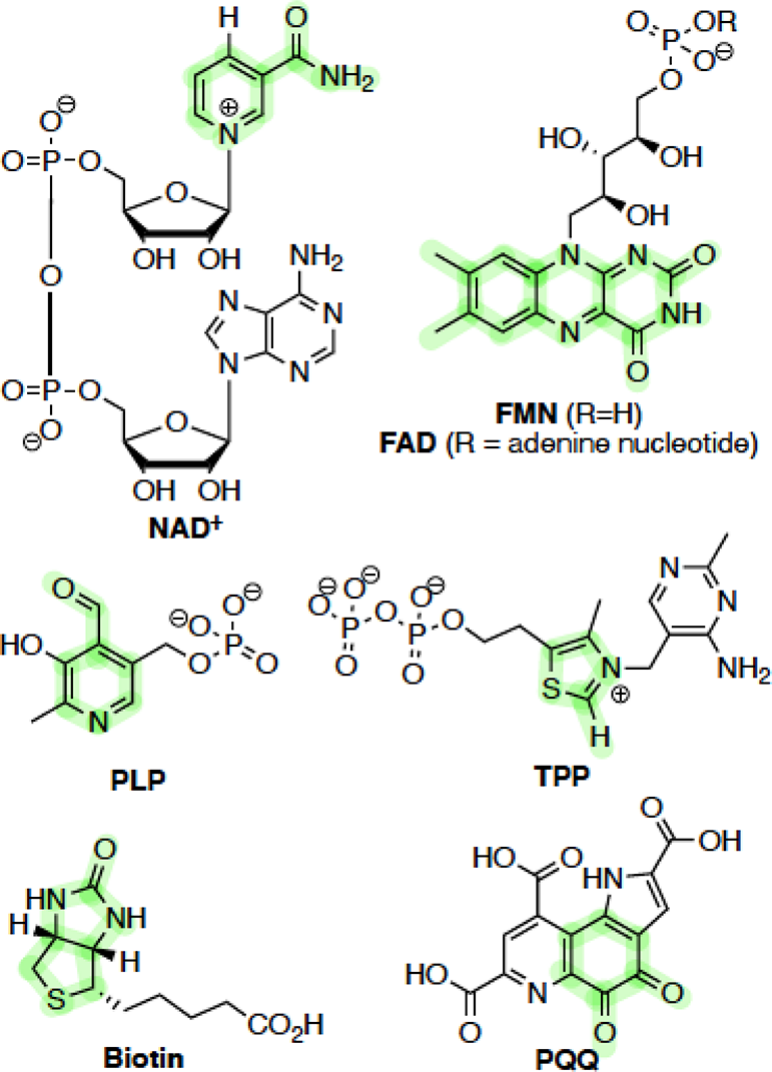
Cofactors That Have Been Used Directly in Organocatalysis and Form the Basis of This Synopsis, with the Key Moieties Highlighted in Green

One significant opportunity for biocatalysis is cofactor engineering, which could involve the use of native or non-native cofactors with enzymes. Enzymes work in tandem with small molecule cofactors, which have intrinsic catalytic properties of their own. Cofactor catalysis provided inspiration for organocatalysis, with a range of the common organocatalysts in modern synthetic chemistry based on cofactor scaffolds derived from biology. Within the bioinformatics database of 27 cofactors, a selection of six have been directly used in organocatalysis to our knowledge (Scheme 2).^14^ The core scaffolds of five cofactors (NADH, FADH, PLP, TPP, PQQ) have been used for organocatalyst design. In the case of the cofactor biotin, organocatalysis has instead employed exceptionally strong binding with streptavidin to exert control on reaction outcome. Chemical intuition has helped modify these cofactors for organocatalytic benefit, leading to enhanced reactivities, stereoselectivities, and reaction scope. Recent explorations in biocatalysis have seen the application of native cofactors with wild-type or mutant enzymes to extend substrate scope and reaction chemoselectivity, and, in fewer cases, enable the catalysis of non-native transformations.

To date, there has been limited exploration of the application of chemically modified (non-native) cofactors in enzymatic biocatalysis, and the area is ripe for future development. In particular, the many recent advances in organocatalysis have resulted in optimized organocatalysts with significant structural differences from their biological cofactor origins, but with enhanced substrate reaction scopes. The combination of chemically modified cofactors with corresponding cofactor-dependent enzymes could provide opportunities for biocatalysis of new-to-nature chemistry with stereocontrol and additional catalytic advantage potentially provided by the enzyme. Furthermore, over the past 5 years, there has been a significant shift toward the use of more sustainable solvents in organocatalysis.^15^ Organocatalysts that function in more polar, aqueous environments are potentially excellent candidates as non-native cofactors for enzymatic biocatalysis.

Within this synopsis, we will focus on the cofactors in Scheme 2, which have been used in organocatalysis. Where examples are available, we discuss the application of chemically modified cofactors in biocatalysis. Our synopsis is directed particularly toward chemists with an emphasis on asymmetric catalysis in line with the focus of the special issue. An excellent recent review in 2022 by Lechner and Oberdorfer, also on cofactor catalysis, covers complementary examples.^16^

Looking to the future, synergistic approaches combining the fundamental knowledge developed in both bio- and organo-catalysis could unveil new areas of asymmetric chemistry, particularly through the introduction of non-native cofactors to biocatalytic transformations to enhance reactivity and scope.

## Nicotinamide Cofactors

**Scheme 3:**
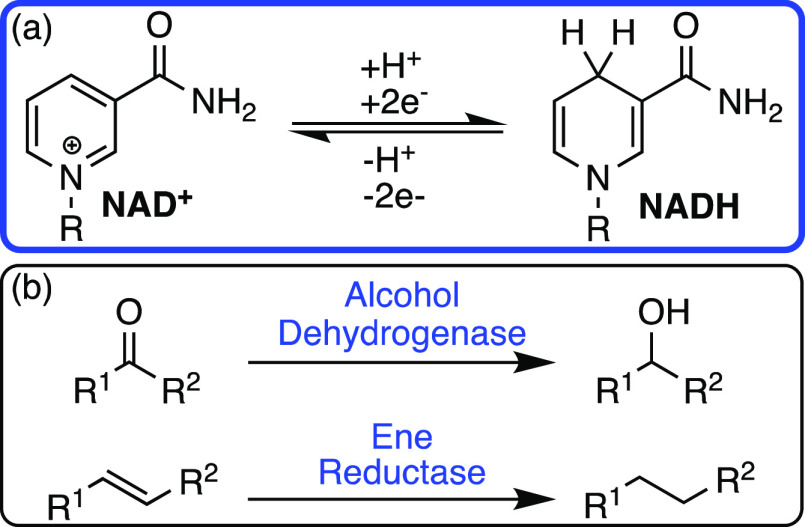
(a) The Key Two-Electron Oxidation/Reduction Reaction of NAD(H) and (b) Native NAD(H)-Dependent Enzymes

Nicotinamide cofactors, NAD^+^/NADH and NADP^+^/NADPH, are responsible primarily for the formal transfer of hydride within biological systems. They consist of two key structural moieties: an electrochemically active nicotinamide and an adenosyl dinucleotide, which differentiate the anabolic (NADP^+^/NADPH) and catabolic (NAD^+^/NADH) pathways. These differences are essential from a cellular perspective but are less relevant from the perspective of biomimetic catalysis.^17^ Within biological systems, NADH couples to a wide range of biologically important enzymes, particularly dehydrogenases and ene-reductases (Scheme 3). During reactions, NADH is used stoichiometrically to transfer hydride to C=X systems (typically, ketones, aldehydes, imines, alkenes) and must be reformed by other reduction processes within the cell.^18^ Typically, this regeneration involves flavin-type cofactors (*vide infra*).

**Scheme 4:**
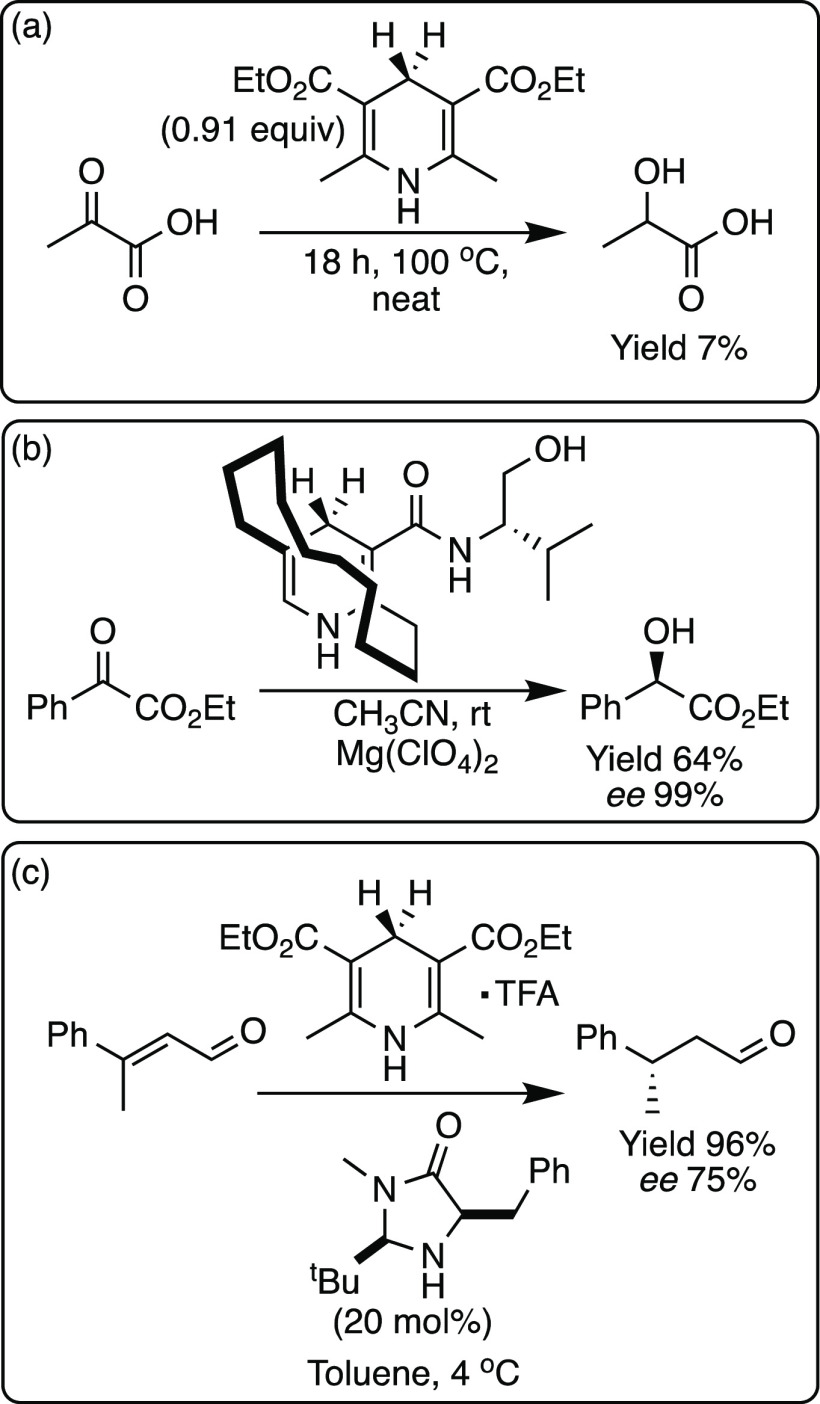
Hantzsch Esters for the Reduction of (a) an α-Keto Acid, (b) an α-Keto Ester, and (c) a Michael Acceptor with Dual Pyrrolidine-Type Enamine Catalysis

NADH was one of the earliest cofactors harnessed by synthetic chemists in the absence of enzymes. Westheimer and Mauzerall showed that Hantzsch esters, dihydropyridine analogues of NADH, could successfully mediate hydride transfer in a metal-free process.^19^ Traditional synthetic protocols for selective reductions utilize homogeneous metal-based catalysts plus hydrogen or hydridic sources with stereoselective ligands. This work opened a field of organocatalysis focused on asymmetric hydrogenations, for the development of stereocenters (Scheme 4).^20,21^ Organocatalytic systems combining both iminium ion catalysis and Hantzsch esters have been developed to give high yielding, enantioselective reductions without the need for metal catalysts (Scheme 4c).^21^

**Scheme 5:**
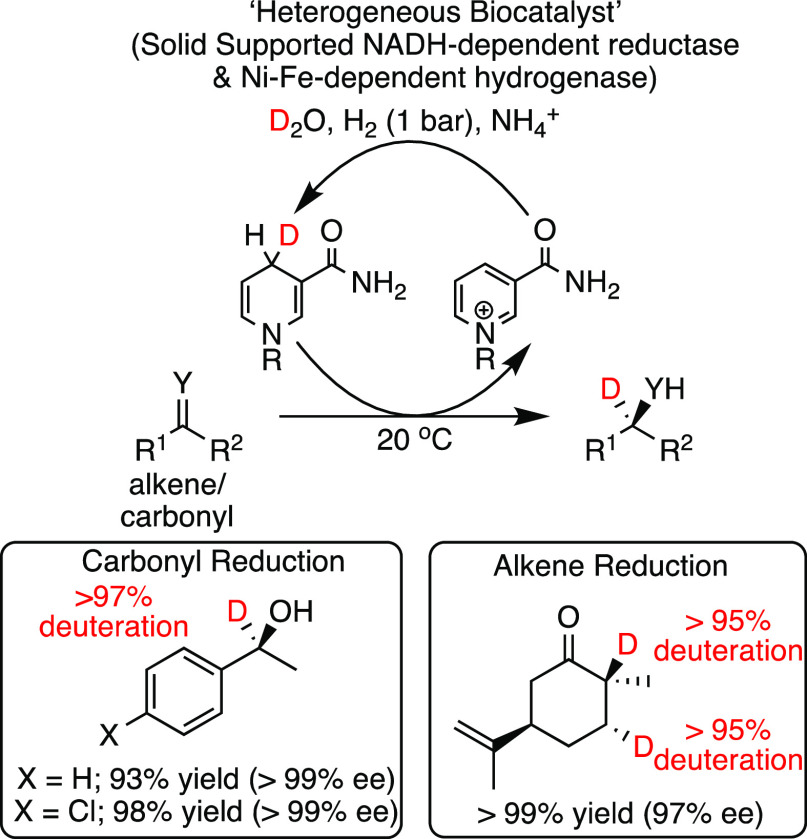
Application of NAD(H) Dependent Enzymes in Biocatalysis for the Deuteration of a Range of Substrates

A recent example of the biocatalytic application of NADH-dependent enzymes employs a deuterated-NADH cofactor for the stereoselective deuteration of a range of alkene and carbonyl systems (Scheme 5).^22^ Pharmaceutically relevant substrates were explored, and excellent yields and stereoselectivities were observed. Biocatalytic deuteration has the advantage of excellent enantioselectivity, with no metal-based catalyst and a relatively inexpensive source of deuterium (D_2_O). The biocatalytic system used a heterogeneous combination of two enzymes immobilized on a solid support including a hydrogenase with a nickel–iron active site and NAD^+^ dependent reductase with a flavin mononucleotide active site. The requirement for a second hydrogenase highlights a key challenge for NADH-biocatalysis: the need for simple, inexpensive techniques for cofactor recycling. In this case, hydrogen gas was employed as the sacrificial reductant.

**Scheme 6:**
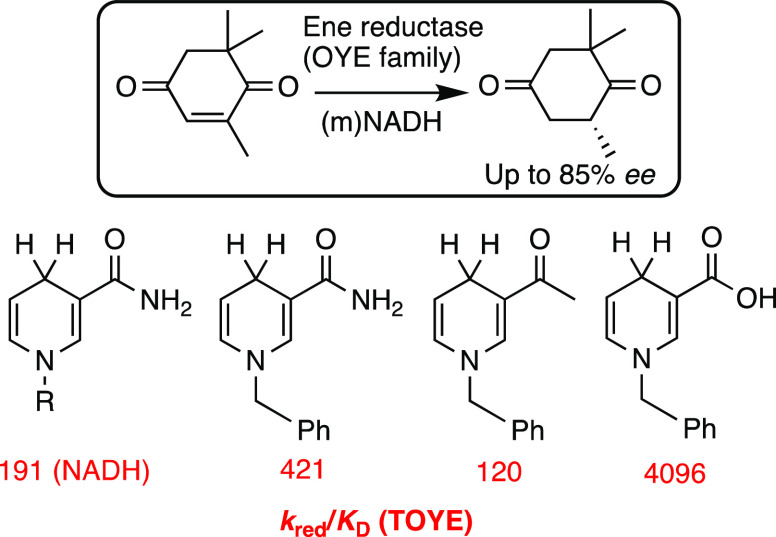
Application of Non-Native NADH Mimics ((m)NADH) in Biocatalytic Reductions Using Ene Reductases from the Old Yellow Enzyme (OYE) Family (TOYE: Thermophilic OYE)

Several NADH mimics were designed and used in tandem with ene reductases from the Old Yellow Enzyme (OYE) family, alongside flavin mononucleotide, to reduce activated alkenes (Scheme 6).^23^ Chemical intuition allowed improvements in performance beyond the natural cofactor (kinetic constants for the hydride transfer, *k*_red_; apparent dissociation constants for flavin reduction, *K*_D_). In addition, biocatalytic, stereoselective epoxidations and sulfoxidations were reported using a flavin cofactor system (with O_2_) in conjunction with a NADH mimic, 1-benzyl-1,4-dihydronicotinamide.^24^ In an earlier report of enzymatic hydroxylation using the same 1-benzyl NADH mimic, the wild-type P450 BM-3 enzyme showed no reaction with a mimic, whereas a double mutant (W1064S/R966D) gave significant enhancement in activity to the same order of magnitude as the native cofactor.^25^ These examples highlight the benefits of synergistic approaches combining key developments in organo- and biocatalysis.

For NADH recycling, catalytic quantities of a rhodium complex were employed, and the complex was recycled using formate.^23,25^ This promising development reduces the amounts of the cofactor hydride donor required for catalytic levels and paves the way for the use of alternative catalysts, potentially using less expensive, less toxic, and more earth abundant metals, in the same recycling role. Continuous flow methods with enzymes immobilized on a solid carbon nanotube support allowed NADH recycling using a hydrogenase/NAD^+^-reductase as the H_2_-driven cofactor recycling system, which significantly reduced waste and the need for the costly, stoichiometric NADH cofactor.^26,27^ Reductions of imine and carbonyl systems gave high conversions and turnover numbers and very high enantiomeric excesses.^26,27^

## Flavin Cofactors

**Scheme 7:**
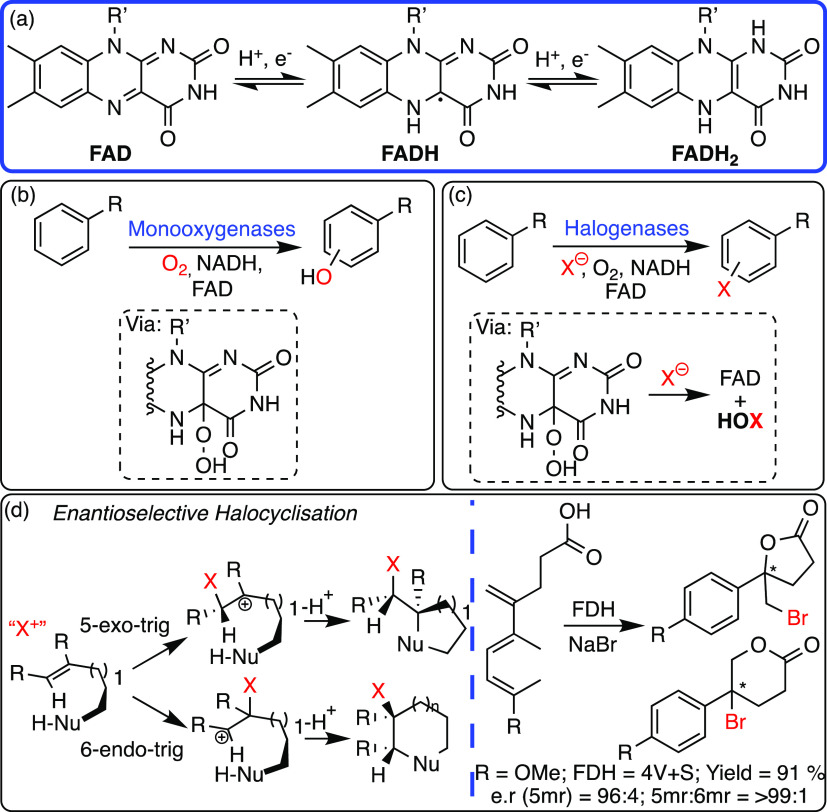
(a) The key Reactions of FAD, Two One-Electron Oxidation/Reductions; (b,c) Native FAD Dependent Enzymes and Flavin Hydroperoxide Intermediates; and (d) Flavin-Dependent Halocyclization Using Native Flavin Cofactor and a Mutant Tryptophan Halogenase Enzyme (FDH = Flavin-Dependent Halogenase) Enantioselective halocyclizations by tryptophan halogenase mutants favor the five-membered ring (5mr) in high e.r.s over the six-membered rings (6mr).

Despite often being used in tandem with NADH in biological systems, flavin cofactors are not limited to hydride/hydrogen transfers. Halogenations, C–C bond formations, and alkene or nitro group reductions are also facilitated and have been reviewed.^28^ Within biological systems, flavin typically has either a mononucleotide (FMN) or adenine dinucleotide (FAD) prosthetic group; however, it is the central flavin moiety which is responsible for the electron transfer properties. Biological applications of flavoenzymes include two-electron oxidations in major metabolic systems, halogenations of major natural products, photorepair of DNA damage, and blue-light sensing cryptochromes.^29^ The key mechanistic step in the majority of flavin-dependent oxidations is formation of the reactive flavin hydroperoxide, which is able to transfer an oxygen atom to a substrate (Scheme 7).^30^ As with NADH, flavin cofactors require regeneration during redox processes, typically via another enzymatic cycle, coupled with the nicotinamide cofactor. This is a key consideration for the development of biocatalytic and organocatalytic processes using flavin mimics.^31^

Frequently, flavin derivatives have been used for the regioselective halogenation of aromatic compounds with inorganic halides via tryptophan halogenases.^32^ This enzymatic strategy often improves selectivity while avoiding the need for typically hazardous chemical halogenating reagents. Moreover, structure guided mutagenesis has allowed the development of enzymes with different regioselectivities and broader substrate scopes with potential for industrial applications.^32^ In a recent development, expanding the scope of flavin-dependent halogenations, a selection of enantioselective non-native halocyclisations highlighted the potential for additional transformations (Scheme 7d).^33^ Previously, these transformations have been carried out with vanadium haloperoxidases; however, mutated flavin-dependent halogenases, normally used in arene halogenations, were demonstrated to perform this enantioselective transformation in good to excellent yields. Mutant enzymes were explored, and while those developed for arene halogenations could also catalyze halocyclisations, the majority of native enzymes could not.^33^

Other biocatalytic applications involve oxidized flavin (FAD) for the synthesis of imines from amines by monoamine oxidases,^34^ the synthesis of enantioenriched amino acids and polymer building blocks using ene reductases,^35^ and Baeyer–Villiger oxidations using Bayer–Villiger monooxygenases and have been reviewed.^31^

**Scheme 8:**
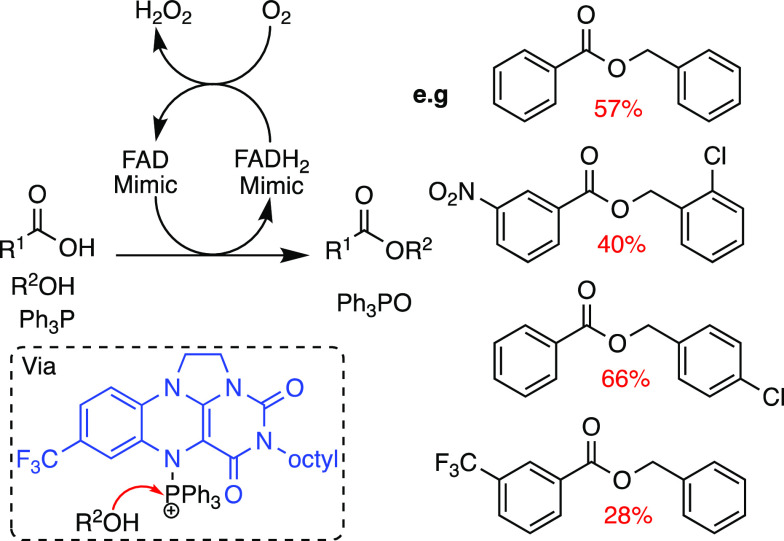
FAD-Catalyzed Mitsunobu Esterification, Which Proceeds via a Flavin (blue)triphenylphosphine Adduct

As organocatalysts, flavin cofactors and derivatives have been used for a range of transformations. The key flavin hydroperoxide intermediate is unstable and requires stabilization within the enzyme active site with specific protein interactions; however, organocatalytic generation of this intermediate has been possible through chemical modifications to flavin. The *N*-alkylation of flavin permits the formation of stable hydroperoxides that can be used for organocatalytic oxidations with inexpensive oxidants.^30^ Moreover, catalytic loadings of alkyl-flavins were possible by introducing hydrazine as the reducing agent to regenerate flavin, before a continued reaction with molecular oxygen to form the active oxidant. In a recent example, the phosphine N(5)-adducts of an alkylated flavin were used to catalyze aerobic Mitsunobu reactions (Scheme 8).^36^ The active Mitsonobu reagent could be regenerated by molecular oxygen as opposed to toxic and explosive azodicarboxylates.^36^

Alongside oxidation reactions, flavin cofactors have been employed in the challenging reductions of carbon–carbon double bonds through the catalytic generation of a diimide reductant from hydrazine. Despite significant interest in flavin-based organocatalysis, examples of enantioselective syntheses are limited. Chiral groups have been employed as flavin N-alkyl/aryl substituents; however, ee’s have not exceeded 65%. Planar chiral aromatic groups have typically been designed to direct the stereo-outcome through hydrophobic π–π interactions.^37^ Alternative approaches to choreograph stereochemistry have used artificial receptors such as cyclodextrins and nonenzymatic proteins that bind to flavin cofactors to access ee’s of up to 80%.^30,38,39^

Most biomimetic analogues of flavin have been developed to stabilize the flavin hydroperoxide intermediate outside of a biological setting. As such, reintroduction of these analogues to enzymes has not been explored. Nevertheless, examples such as the bridged N-alkyl flavin derivative used to catalyze the Mitsunobu reaction (Scheme 8)^36^ could be considered in a chiral biocatalytic enzyme environment to develop new asymmetric biotransformations.

## Pyridoxal Phosphate (PLP)

**Scheme 9:**
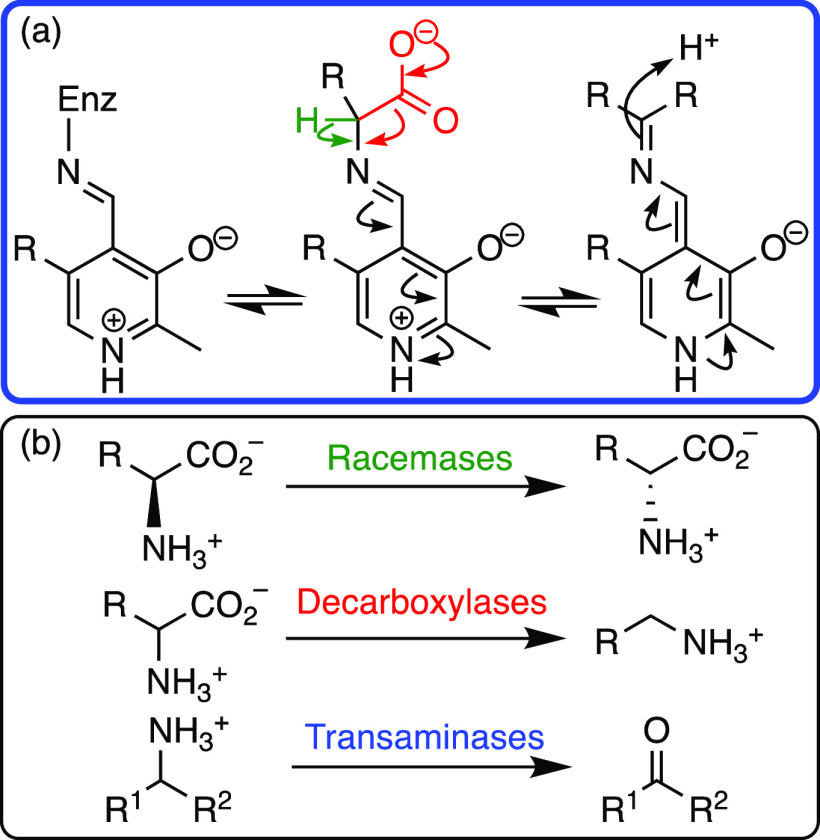
(a) Schiff Base Formation with an Amino Acid Substrate Facilitating Deprotonation or Decarboxylation and (b) Native PLP Dependent Enzymes

PLP is a cofactor for a broad range of enzyme-catalyzed reactions of amino acids, reducing the activation barrier for the formation of α-carbanions. Mechanistically, a lysine residue within the enzyme active site forms a Schiff base, or iminium ion, with PLP. Upon binding of amino acid, the enzyme-cofactor iminium ion converts to a PLP-substrate iminium ion, providing activation toward a variety of transformations including decarboxylation, racemization, and transamination (Scheme 9).^40^ In a model study to quantitatively evaluate enzyme-PLP catalysis of amino acid racemization, it was shown that formation of an iminium ion with acetone reduces the C(α)-H p*K*_a_ by seven units.^41^

Biological PLP-dependent transformations have inspired two fields of organocatalysis: reactions of carbonyl compounds catalyzed by amines and reactions of amines catalyzed by aldehydes.^42,43^ The latter is most similar to PLP catalysis, and various analogues have been explored. However, both areas are broadly classed within enamine and iminium ion catalysis. Development of PLP biomimetics is challenging owing to the need to meet three criteria: (1) the carbonyl component of the cofactor mimic must have significant electron-withdrawing character to activate the α-position of the amine toward deprotonation; (2) the catalyst-substrate iminium ion must be less reactive than the reacting electrophile at the α-amino position; (3) the carbonyl catalyst must direct the approaching electrophile for asymmetric reactions.^42,44^

**Scheme 10:**
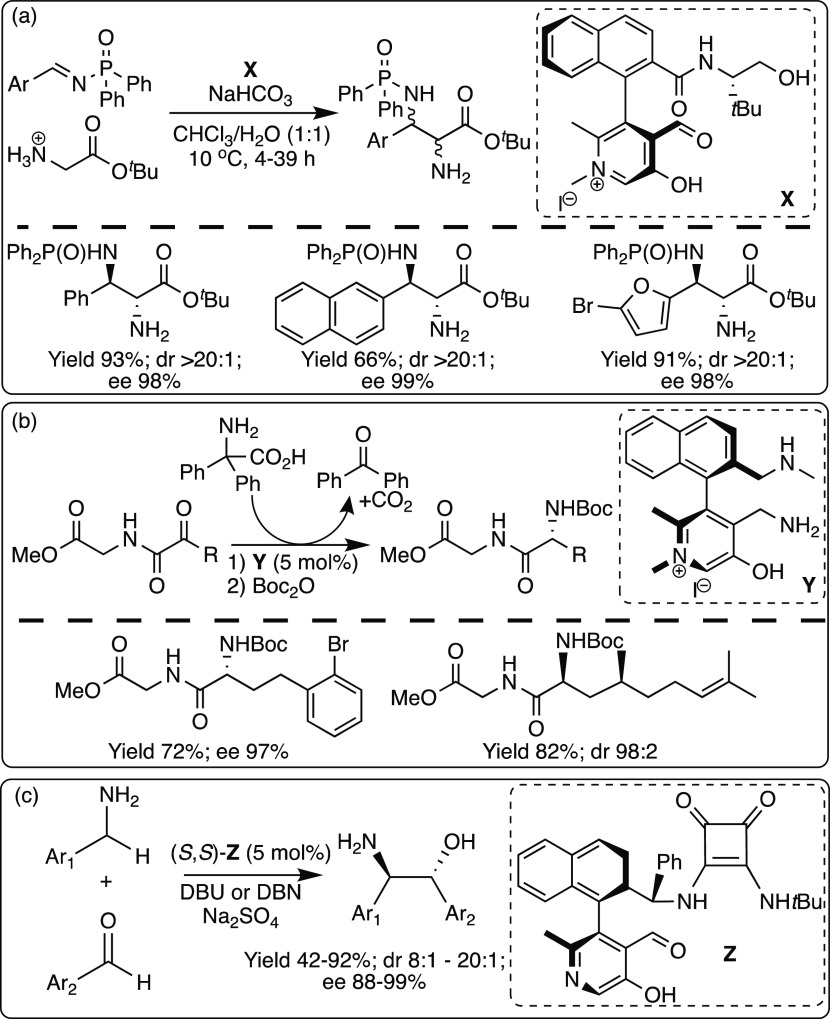
Asymmetric Organocatalytic Transformations Catalyzed by Mimics of PLP: (a) Mannich Reaction between Glycinate and N-Diphenylphosphinyl Imines; (b) Transamination; and (c) α C(sp^3^)–H Addition of Benzylamines to Aldehydes

A biomimetic chiral PLP derivative has been developed to catalyze an asymmetric Mannich reaction between glycinate and N-diphenylphosphinyl imines (Scheme 10a). Reactions were high yielding with excellent enantioselectivities and substrate scope.^44^ The products can readily be deprotected to yield chiral diamines in high yields, dr’s, and ee’s. A chiral pyridoxamine catalyst has also been developed for biomimetic, asymmetric transaminations, providing a novel approach to the synthesis of complex peptides (Scheme 10b).^45^ Recently, a chiral pyridoxal catalyzed asymmetric α C(sp^3^)–H addition of NH_2_-unprotected benzylamines to aldehydes was reported, providing a straightforward approach for the synthesis of chiral β-aminoalcohols with excellent dr’s and ee’s (Scheme 10c).^46^ In addition, there has been significant exploration of aldehyde catalysis for a range of transformations including hydroaminations, hydrations, and hydrolyses. Generally, the aldehyde catalysts are not directly based on the structure of PLP, instead linking chiral groups to an aldehyde; however, their modes of action are similar: activating the α-amino position to perform challenging transformations.^47^

In a biocatalysis context, PLP-dependent enzymes using a native cofactor have been used to catalyze the formation of a range of pipecolate structures (derivatives of piperidine-2-carboxylic acid), which are challenging to synthesize chemically, yet important within the pharmaceutical industry.^48^ Transaminases are particularly useful biocatalysts for accessing synthetically challenging and expensive chiral amine building blocks.^49^ Several ω-transaminases have good substrate scope and can accept aliphatic amines and ketones alongside amino and keto acids.^50^ In nature, transaminases are generally (*S*)-selective; however, engineering techniques have allowed access to some (*R*)-selective systems with increased substrate scopes.^51−53^ A notable example of their application is the large scale production of an antidiabetic drug, Sitagliptin.^54^ Other PLP-dependent enzymes have also been used for important chemical transformations, such as lysine decarboxylase for the synthesis of cadaverine, a useful building block for the polymer industry; threonine aldolase for the synthesis of β-hydroxy-α-amino acids, core building blocks in many antibiotics and immunosuppressants; and cystathionine β-lyase for the synthesis of volatile sulfur-containing compounds for the food and fragrance industry.^49^

Despite the broad exploration of PLP-dependent biocatalysis, there has been limited exploration of non-native reactions and cofactors, such as the utilization of the modified cofactor analogues successful in organocatalysis, in enzymatic biocatalysis with PLP-dependent enzymes. Perhaps the broadness of reactivity and scope of PLP-dependent enzymes limits the need for such exploration; however, the ability to carry out challenging transformations on industrial scales in a sustainable manner warrants an exploration of PLP-dependent enzymes in non-native environments with non-native cofactors.

## Biotin

**Scheme 11:**
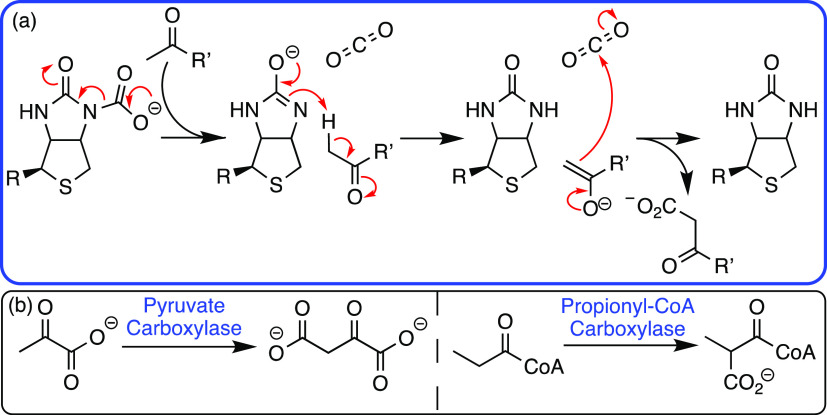
(a) The Key Reaction of Biotin in Biotin-Dependent Enzymes and (b) Native Biotin Dependent Enzymes Including Pyruvate and Propionyl Carboxylases

Biotin is synonymous with the formation of very tight binding streptavidin–biotin host–guest complexes, which have been utilized widely for affinity capture purposes. As an enzyme cofactor, it is covalently bound by an amide bond, via its valerate side chain, to carboxylase enzymes. It transfers carbon dioxide via two half-reactions (Scheme 11) across three classes of biotin-dependent enzymes: carboxylases (class I), decarboxylases (class II), and transcarboxylases (class III). Eukaryotic biotin-dependent enzymes are all class I and include pyruvate carboxylase (gluconeogenesis), propionyl-CoA carboxylase (odd-chain fatty acid synthesis), and acetyl-CoA carboxylase (fatty acid synthesis).^55^ Mechanistically, it is proposed that the covalent adduct of biotin and carbon dioxide cleaves, resulting in an anionic intermediate at the urea moiety of biotin. Subsequently, enolization of a substrate such as pyruvate, by the biotin intermediate, results in a nucleophile capable of reacting with the released carbon dioxide).^55^

Despite the transfer of carbon dioxide being a useful transformation within synthetic chemistry, to our knowledge there are no examples of organocatalytic carboxylation using biotin or biotin mimics. Instead, organocatalysis has taken advantage of the very tight binding within biotin–streptavidin complexes to introduce enzyme-like control on the outcomes of different organocatalytic reactions. Several transformations have been explored, including iminium ion catalysis in the reaction of cinnamaldehyde with nitromethane,^56^ enamine-catalyzed aldol reactions,^57^ anion−π catalysis,^58^ and organocatalytic transfer hydrogenations,^59^ which have been reviewed.^16^ The supramolecular streptavidin host, with inherent asymmetry, imparts good diastereo- and enantioselectivities.

## Thiamine Pyrophosphate

**Scheme 12:**
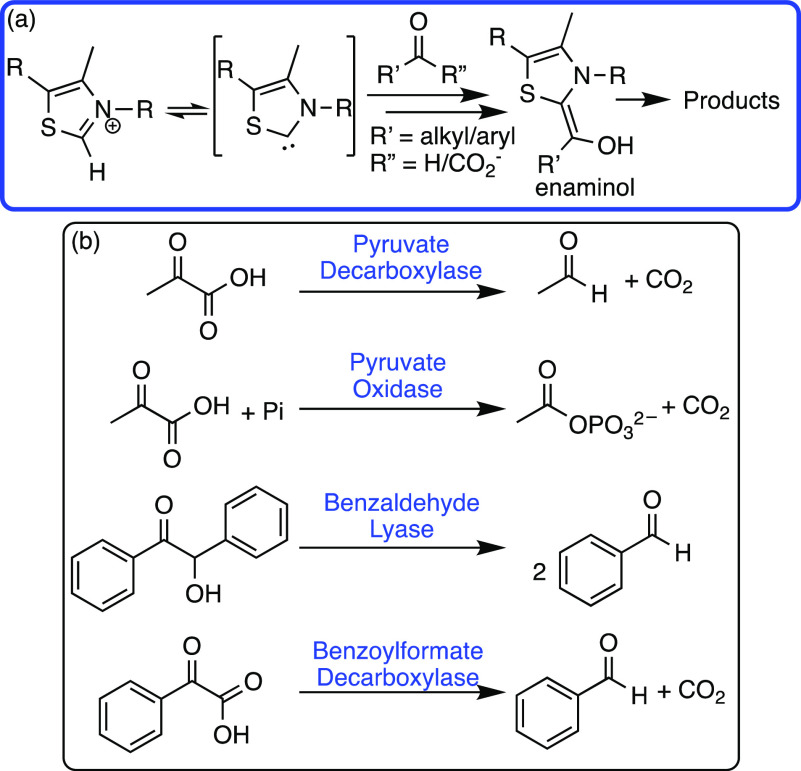
(a) The Key Reaction in TPP-Dependent Enzymes and (b) Native TPP-Dependent Enzymes

Perhaps one of the most well-known cofactors among chemists is thiamine pyrophosphate (TPP) owing to its pivotal role in the development of N-heterocyclic carbene (NHC) organocatalysis. TPP is a cofactor for many C–C bond making and breaking enzymatic reactions.^60^ Based on the enhanced *C*(2)-H acidity of the key thiazolium moiety of TPP, Breslow proposed a mechanism involving an initial deprotonation step to an NHC followed by reaction with the substrate via an enaminol-type intermediate (Scheme 12).^61^

**Scheme 13:**
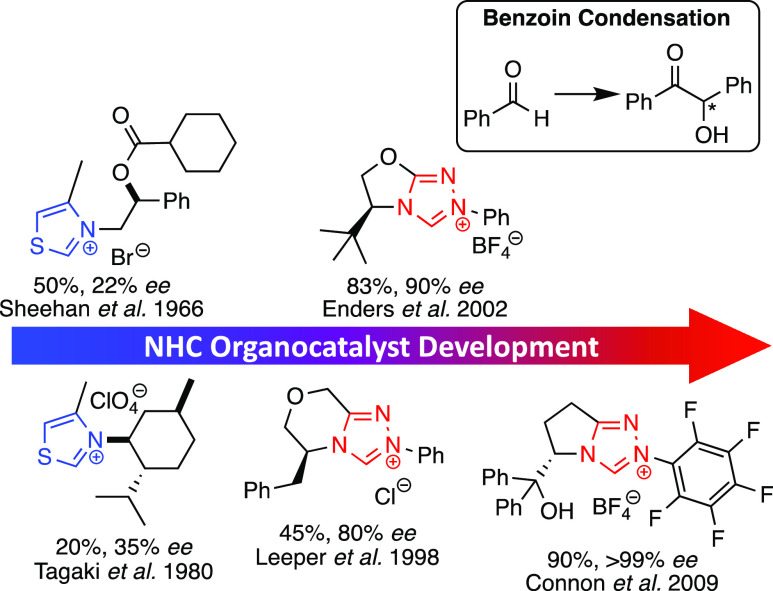
Development of NHC-Organocatalysts from Thiazolium-Derived TPP Mimics Towards 1,2,4-Triazolium Analogues (Yields and ee’s for Model Benzoin Reaction, Inset)

Subsequent screening of a broad range of related heterocyclic azolium catalysts demonstrated that 1,2,4-triazolium salts were generally the most active and stereoselective of the range of N-heterocycles explored as independent organocatalysts (Scheme 13).^3,62−68^ The increased acidity^69^ and added propensity for functionalization in 1,2,4-triazolium ions just one atom removed from the reactive center are beneficial for asymmetric organocatalysis. Finally, the higher activities provide effective catalysis of more complex non-native chemistries such as the intermolecular hydroacylation of unactivated alkenes.^70,71^

**Scheme 14:**
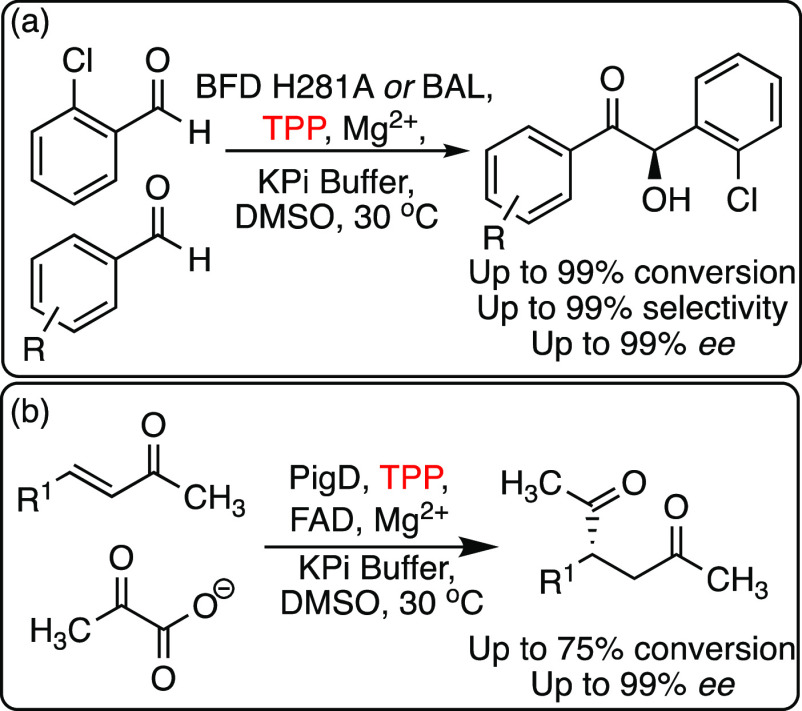
(a) Highly Selective Crossed Benzoin Condensation Catalyzed by TPP-Dependent Enzymes with Native Cofactor Including Benzoyl Formate Decarboxylase (BFD) or Benzaldehyde Lyase (BAL) and (b) Biocatalytic Stetter Reaction Catalyzed by a TPP-Dependent Enzyme (PigD)

Enzymatic biocatalysis with TPP-dependent enzymes has been limited to the native TPP cofactor to date. For example, enzymatic stereo- and chemoselective cross-benzoin reactions could be achieved with different TPP-dependent enzymes with substrate scope extended to a range of alkyl and aryl aldehydes (Scheme 14).^72,73^ Although yields and stereoselectivities for the enzyme-catalyzed benzoin condensations were impressive, those observed for the related Stetter reaction of aldehydes with α,β-unsaturated carbonyl acceptors were lower.^74^ This perhaps highlights the limits of the thiazolium core of the native cofactor and presents opportunities for the more acidic, non-native 1,2,4-triazolium cofactor mimic for asymmetric enzymatic biocatalysis reactions.

## Quinone-Like Cofactors

**Scheme 15:**
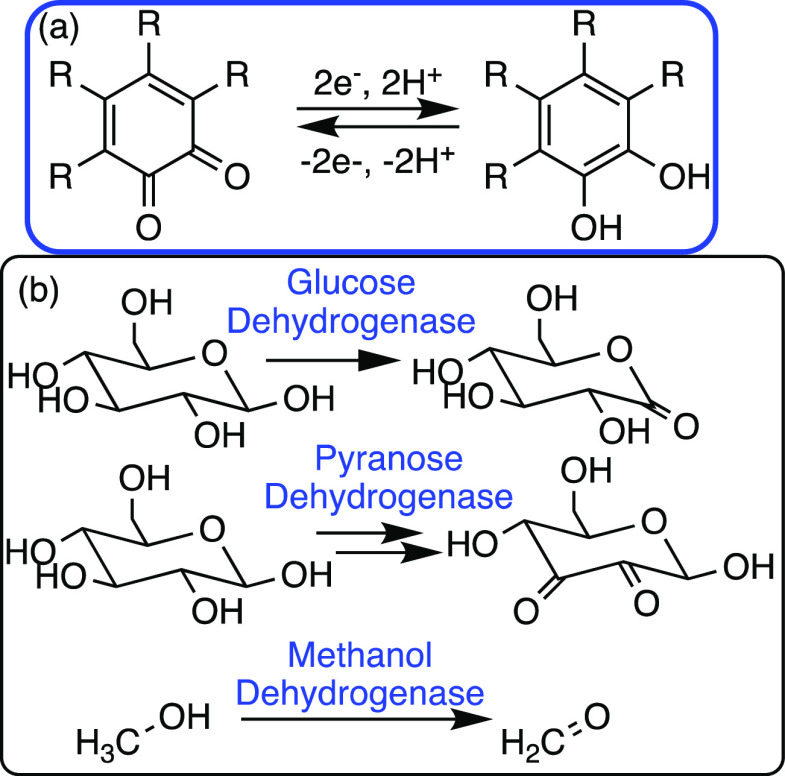
(a) The Key Reaction of Quinones in Quinone-Dependent Enzymes and (b) Native PQQ-Dependent Enzymes

Although this cofactor class is less well-studied to date, orthoquinones such as pyrroloquinoline quinone (PQQ) have received the most attention. PQQ is a highly multifunctional cofactor, which binds to enzymes by electrostatic interactions, and shows excellent redox cycling properties (Scheme 15).^75^

In organocatalysis, several quinone-based catalysts have been explored in transformations such as the aerobic C–H oxidation of amines to imines.^76^ To our knowledge, there has been more limited exploration of quinone-dependent enzymes in biocatalysis or of quinone-based organocatalysts as non-native cofactors. This is likely due to a greater focus on NADH/FAD redox processes. However, this area is ripe for future exploitation to harness and tailor biological processes toward the needs of chemical processes. Previous reviews of PQQ-dependent enzymes have highlighted their potential applications in biocatalysis.^75^

## Conclusions

We have used selected recent examples of synthetic applications of native cofactors and non-native cofactors (cofactor mimics) within biocatalysis and organocatalysis to highlight the potential to exploit advantages and synergies in these areas. Although opportunities for biocatalysis of new-to-nature chemistry are plentiful, there are challenges to address, such as cofactor recycling. Enzymes work in aqueous environments, which supports sustainability and reduces solvent waste. Practically, some chemical feedstocks and downstream products are not water-soluble, and so either surfactants or mixed solvent systems may be required. Managing the mixed waste streams from these processes can be more complex than managing organic solvent waste.

To further develop non-native cofactors, novel synthetic strategies are required; however, the potential to access a broader range of chemical transformations justifies their exploration. Although cofactor mimics developed as organocatalysts retain key structural moieties from the native progenitors, simplifications such as the removal of pyrophosphates to facilitate synthesis and organic solvent solubility potentially reduce binding affinities toward cognate enzymes. Future syntheses may require their reintroduction to the modified cofactor scaffolds. Additionally, directed evolution approaches and targeted protein engineering, combined with structural biology-based modeling, may allow reoptimization of protein structure to accommodate modified cofactor scaffolds.

Traditional syntheses of chiral organocatalysts can be challenging, owing to the need to introduce specific chiral moieties and separate stereoisomers. The addition of chiral groups also often comes at the expense of solubility in polar, “greener” solvents such as water. In contrast, synthetic access to similar achiral non-native cofactors is more straightforward, and these could be used in tandem with cofactor dependent enzymes, or mutants thereof, to afford enzyme-based stereocontrol of the reaction outcome.

The significant advantages to exploring the areas of non-native cofactor biocatalysis warrant investigation. For a sustainable (bio)chemical future, there is no “one size fits all” answer, and diverse catalytic solutions are needed.

## Data Availability

The data underlying this study are available in the published article.
